# Age-Associated Hyper-Methylated Regions in the Human Brain Overlap with Bivalent Chromatin Domains

**DOI:** 10.1371/journal.pone.0043840

**Published:** 2012-09-24

**Authors:** Corey T. Watson, Giulio Disanto, Geir Kjetil Sandve, Felix Breden, Gavin Giovannoni, Sreeram V. Ramagopalan

**Affiliations:** 1 Department of Biological Sciences, Simon Fraser University, Burnaby, British Columbia, Canada; 2 Wellcome Trust Centre for Human Genetics, University of Oxford, Oxford, United Kingdom; 3 Department of Clinical Neurology, University of Oxford, The West Wing, John Radcliffe Hospital, Oxford, United Kingdom; 4 Department of Informatics, University of Oslo, Blindern, Oslo, Norway; 5 Blizard Institute, Queen Mary University of London, Barts and The London School of Medicine and Dentistry, London, United Kingdom; 6 Medical Research Council Functional Genomics Unit, Department of Physiology, Anatomy & Genetics, University of Oxford, Oxford, United Kingdom; RWTH Aachen University Medical School, Germany

## Abstract

Recent associations between age-related differentially methylated sites and bivalently marked chromatin domains have implicated a role for these genomic regions in aging and age-related diseases. However, the overlap between such epigenetic modifications has so far only been identified with respect to age-associated hyper-methylated sites in blood. In this study, we observed that age-associated differentially methylated sites characterized in the human brain were also highly enriched in bivalent domains. Analysis of hyper- *vs*. hypo-methylated sites partitioned by age (fetal, child, and adult) revealed that enrichment was significant for hyper-methylated sites identified in children and adults (child, fold difference = 2.28, *P* = 0.0016; adult, fold difference = 4.73, *P* = 4.00×10^−5^); this trend was markedly more pronounced in adults when only the top 100 most significantly hypo- and hyper-methylated sites were considered (adult, fold difference = 10.7, *P* = 2.00×10^−5^). Interestingly, we found that bivalently marked genes overlapped by age-associated hyper-methylation in the adult brain had strong involvement in biological functions related to developmental processes, including neuronal differentiation. Our findings provide evidence that the accumulation of methylation in bivalent gene regions with age is likely to be a common process that occurs across tissue types. Furthermore, particularly with respect to the aging brain, this accumulation might be targeted to loci with important roles in cell differentiation and development, and the closing off of these developmental pathways. Further study of these genes is warranted to assess their potential impact upon the development of age-related neurological disorders.

## Introduction

Epigenetic modifications, such as DNA methylation and post-translational modifications of histone proteins, are indispensable for many aspects of genome function, including gene expression. DNA methylation patterns at specific CpG-sites can vary over time within an individual and correspondingly, age-related methylation changes have been identified in multiple tissues and organisms [1]–[8]. A recent study of changes in DNA methylation in blood cells associated with aging revealed an enrichment of differentially-methylated sites, specifically those exhibiting increased methylation with age (hyper-methylation), in bivalently marked regions of the genome marked by the presence of both trimethylated lysine 4 and 27 in histone H3 (H3K4me3 and H3K27me3 respectively)[3]. Due to the predominant effect of H3K27me3, bivalent domains are known to take on repressed states while remaining ‘poised’ for active gene expression, a feature important to cell pluripotency and differentiation [9], [10].

Interestingly, the accumulation of DNA methylation at bivalent domains has been proposed to impact gene expression programs associated with both cell differentiation and carcinogenesis [11]. Given the relationship between cancer and aging, the finding that age-associated hyper-methylation has been shown to preferentially occur within bivalent domains is intriguing and warrants further study [3], and also suggests that the build up of DNA methylation at bivalent domains with age may occur in additional tissues with potential roles in the pathogenesis of other age-related disorders. In the current study, we sought to build upon our understanding of age-associated DNA methylation by investigating whether recently characterized age-associated DNA hyper- and hypo-methylated loci in the human brain [8] had a similar relationship with bivalently marked chromatin domains in human embryonic stem cells [12].

## Methods

### Data acquisition and mining

Raw association data, including *P*-values for 27,578 genome-wide CpG sites from [8], were kindly provided by the authors of the study; these methylation data were generated from human brain samples (dorsolateral prefrontal cortex) of 108 subjects using the Illumina Infinium HumanMethylation27 BeadChip [8]. For the current study, a Bonferonni corrected *P*-value (1.81304E-06) was applied to determine CpG sites with the most significant age-associated methylation changes within each of three age groups, as defined by Numata et al. [8]: fetal, child (0–10 years of age), and adult (>10 years of age). After applying this correction, sites were first partitioned into hypo-*vs*. hyper-methylated sites; hyper-methylated sites were defined as those with slopes >0.0 (*i.e*., those at which methylation increased with age), whereas hypo-methylated sites were defined as those with slopes <0.0. Data were then further partitioned into additional subsets: (1) all significant sites for each age group (using *P*-value <1.81304E-06), (2) significant sites unique to each age group (*i.e*., those sites with significant *P*-values <1.81304E-06 in a given age group that were not also significant (*P*-value >1.81304E-06) in either of the other two age groups), and (3) the top 100 most significant non-overlapping sites per age group. In addition, CpG sites with reported age-related changes in methylation identified in blood were also used [3], [4], [7], including aging-associated differentially methylated sites from samples of patients with type-1 diabetes [4]. When necessary, differentially methylated sites from these studies were also designated as either hyper- or hypo-methylated. Coordinates of CpG probe IDs from brain and blood studies were considered as single points in analyses below, based on the first base pair position of each probe. For bivalent chromatin sites, gene coordinates (+−2 KB) of 1,792 previously identified genes associated with co-methylation of H3K4 and H3K27 in human hES3 cells [12] were used. Probe and gene coordinates were based on the hg18 (NCBI 36) human genome reference build.

### Data Analysis

We used the Genomic Hyperbrowser (http://hyperbrowser.uio.no/hb/)[13] to perform all analyses on genome coordinates. Enrichment was defined as the ratio of the proportion of probe coordinates covered by bivalent gene regions, to the proportion of non-probe coordinates covered by bivalent gene regions. The significance of overlap was tested at a whole genome scale, by asking whether age-associated probe coordinates fell within bivalent gene coordinates more often than expected by chance; for this, we defined a null model in which probe coordinates were preserved exactly, and where the positions of bivalent gene coordinates were randomized while preserving the observed distribution of lengths and distances between these bivalent gene coordinates. The base pair overlap between the two tracks was calculated for the real data, as well as for 50,000 Monte Carlo samples from the null model. *P*-values were calculated as the proportion of Monte Carlo samples being equal to or more extreme than the observed overlap.

For comparisons of the degree of overlap observed between bivalent gene loci and different subsets of age-associated differentially methylated sites (*e.g*. adult brain hyper- *vs*. hypo-methylated sites), case-control tracks were created for each pair of subsets. In each instance, case and control tracks consisted of probe coordinates unique to either of the two subsets being compared. For this analysis, Monte Carlo simulations (n = 50,000) were performed in which case-control labels for each of the coordinates were permuted randomly. *P*-values were calculated by comparing the observed base pair overlap between case tracks and bivalent regions against the corresponding distribution for 50,000 Monte Carlo samples. The fold enrichment difference in overlap between case and control tracks was calculated as the ratio between the proportions of case and control tracks that overlapped with bivalent regions.

**Table 1:** Global enrichment of differentially methylated loci in bivalent chromatin domains.

	Global Enrichment	
	bivalent sites (n = 1792)	*P* value
**Brain Adult (n = 2756)** [8]	3.063	**0.00002**
**Brain Child (n = 888)** [8]	2.814	**0.00002**
**Brain Fetal (n = 125)** [8]	0.7914	0.7809
**Brain Adult only (n = 2307)** [8]	3.066	**0.00002**
**Brain Child only (n = 441)** [8]	2.616	**0.00002**
**Brain Fetal only (n = 103)** [8]	0.8008	0.7491
**Rakyan et al. ** [**3**] ** (n = 131)**	12.86	**0.00002**
**Alisch et al. ** [**7**] ** (n = 2026)**	2.09	**0.00002**
**Teschendorff et al. ‘S3’ ** [**4**] ** (n = 583)**	3.495	**0.00002**
**Teschendorff et al. ‘S5’ ** [**4**] **, T1D (n = 683)**	5.294	**0.00002**

We used DAVID [14], [15] to assess the biological function of genes associated with hyper-methylated sites unique to adult brain [8], considering those that overlapped bivalent sites compared to those that did not [12]. Results for gene ontology were assessed using the Functional Annotation Tool (GOTERM_BP_FAT).

## Results

### Enrichment of age-associated differentially methylated sites in bivalent chromatin domains

Of the 27,578 CpG sites investigated across three age groups, fetal, child, and adult, Numata et al. [8] identified 865, 5,506, and 10,578 loci, respectively, that exhibited age-associated changes in DNA methylation based on a false discovery rate of 5%. To further refine this data set, we applied a Bonferonni correction to the uncorrected data, and compiled a list of 125 fetal, 888 child, and 2,756 adult sites, with the most shared sites between children and adults (child/adult, n = 439; child/fetal, n = 12; fetal/adult, n = 14). After removing shared loci between age groups, there were a total of 2,851 non-overlapping sites (fetal, n = 103; child, n = 441; adult, n = 2,307; Table 1).

**Table 2:** Comparing global enrichment of hypo- vs. hyper-methylated loci in bivalent chromatin domains.

	Global Enrichment		Is overlap for hyper sites > hypo sites?	
	bivalent sites (n = 1792)	*P* value	Fold difference	*P* value
**Brain Adult only, hypo (n = 440)** [8]	0.9055	0.752		
**Brain Adult only, hyper (n = 1867)** [8]	3.659	**0.00002**	4.727	**0.00004**
**Brain Child only, hypo (n = 154)** [8]	1.326	0.2618		
**Brain Child only, hyper (n = 287)** [8]	3.392	**0.00002**	2.28	**0.00164**
**Brain Fetal only, hypo (n = 83)** [8]	0.3875	0.9572		
**Brain Fetal only, hyper (n = 20)** [8]	2.77	0.1299	4.446	0.09896
**Brain Adult only, hypo (top 100)** [8]	0.654	0.8946		
**Brain Adult only, hyper (top 100)** [8]	8.085	**0.00002**	10.67	**0.00002**
**Brain Child only, hypo (top 100)** [8]	1.365	0.2754		
**Brain Child only, hyper (top 100)** [8]	4.427	**0.00006**	2.75	0.05192
**Blood Adult hypo (n = 360)** [4]	2.017	**0.00094**		
**Blood Adult hyper (n = 223)** [4]	6.498	**0.00002**	2.571	**0.00002**
**Adult T1D, hypo (n = 278)** [4]	1.365	0.1393		
**Adult T1D, hyper (n = 405)** [4]	9.255	**0.00002**	4.637	**0.00002**

We tested for enrichment of brain DNA methylation sites identified in each age group with bivalent chromatin domains. We did this by first considering all age-associated sites in each age group (Table 1); both adult and child sites showed significant enrichment in bivalent gene regions (adult, enrichment = 3.06, *P* = 0.00002; child, enrichment = 2.81, *P* = 0.00002), whereas the enrichment of fetal sites in bivalent gene loci was non-significant (enrichment = 0.79, *P* = 0.78). Global enrichment values changed only slightly when sites unique to each age group were tested (adult, enrichment = 3.07, *P* = 0.00002; child, enrichment = 2.62, *P* = 0.00002; fetal, enrichment = 0.80, *P* = 0.75; Table 1).

As a point of comparison, we also tested for overlap between bivalent gene regions and age-associated differentially methylated sites identified in blood samples from three independent studies [3], [4], [7]. Two of these used samples collected from adults [3], [4], and the third was based on sampling from two child cohorts [7]. Data from Rakyan et al. [3] included only hyper-methylated sites identified in blood that overlapped those identified in CD4^+^ T cells and CD14^+^ monocytes. In all cases, sites identified from each of these three studies showed significant overlap with bivalent gene regions (Table 1). The highest enrichment value (12.86; *P* = 0.00002) was observed for sites identified in Rakyan et al. [3], which had previously been analyzed with respect to their overlap with bivalent sites identified in Zhao et al. [12], and was the motivation for this study. We also tested for overlap of bivalent gene regions and 683 differentially methylated sites identified in a cohort of T1D patients [4] and observed a significant global enrichment value of 5.29 (*P* = 0.00002).

### Increased enrichment of hyper- vs. hypo-methylated sites in bivalent genes

Given the high degree of overlap between bivalent loci and sites identified by Rakyan et al. [3](Table 1), which consisted of the top 131 most significantly differentiated hyper-methylated sites associated with age in their samples, we attempted to further partition age-associated sites in brain to better match this dataset. We first partitioned data from Numata et al. [8] by hyper- *versus* hypo-methylated sites unique to each age group and tested these for enrichment in bivalent gene regions. In child and adult age groups, hyper-methylated sites were enriched in bivalent gene regions, whereas hypo-methylated sites were not (Table 2). In addition, the overlap of hyper-methylated sites with bivalent gene regions was significantly greater than hypo-methylated sites in both children and adults (child, fold difference = 2.28, *P* = 0.0016; adult, fold difference = 4.73, *P* = 0.00004; Table 2). A similar trend was noted for fetal-only sites, although the difference between hypo- and hyper-methylated sites was not significant (fold difference = 4.44, *P* = 0.099). A significant difference between the overlap of bivalent genes observed for hyper- and hypo-methylated sites in adult blood [4] was also observed (hyper>hypo, fold difference = 2.57, *P* = 0.00002; Table 2). We next analyzed only the top 100 methylated brain sites unique to child and adult age groups, partitioned by hyper- and hypo-methylated sites. Again, hyper-methylated sites showed a greater overlap with bivalent genes, but this difference was only significant for adult sites (child, fold difference = 2.75, *P* = 0.052; adult, fold difference = 10.67, *P* = 0.00002; Table 2). In addition, the top 100 adult hyper-methylated sites in brain had a higher enrichment value than when all unique sites were considered (enrichment = 8.09, *P* = 0.00002; Table 2), more similar to that observed for the Rakyan et al. [3] dataset (enrichment = 12.86, *P* =  *P* = 0.00002; Table 1). Also striking, was that when aging associated T1D sites [4] were partitioned based on hypo- *vs*. hyper-methylated changes, hyper-methylated sites also exhibited a much higher enrichment compared to hypo-methylated sites (fold difference = 4.64, *P* = 0.00002; Table 2).

### Functional characterization of hyper-methylated adult brain/bivalent genes

We next sought to determine whether there were differences in biological function of adult brain hyper-methylated genes that overlapped bivalent loci compared to those that did not overlap bivalent loci. Of the 1,867 brain hyper-methylated sites unique to adults, 353 overlapped bivalent loci, corresponding to 304 non-redundant genes. The 1,514 hyper-methylated sites that did not overlap bivalent loci corresponded to 1,410 non-redundant genes. A qualitative comparison of the top ten most significant biological functions of these two groups of genes is shown in Table 3. Not surprisingly, given that bivalent domains are known to associate with genes involved in development, eight of the ten biological functions identified for adult brain hyper-methylated/bivalent sites shared the parent GO term “developmental process” (GO:0032502). Notably, among these was the term “neuron differentiation” (4.90×10^−13^; GO:0030182), which is defined as “the process in which a relatively unspecialized cell acquires specialized features of a neuron”. In contrast, none of the biological functions identified for those sites outside of bivalent domains were associated with GO:0032502; in addition, only four of these had *P*-values <0.05.

**Table 3:** Gene ontology results for brain hyper-methylated sites inside and outside bivalent chromatin domains.

Top ten biological functions for brain hyper-methylated adult-only sites overlapping bivalent genes		
GO Term (biological function)	# of genes	Benjamini *P* value
pattern specification process	41	1.30×10^−21^
embryonic morphogenesis	41	1.40×10^−19^
regionalization	31	2.90×10^−16^
regulation of transcription, DNA-dependent	89	2.90×10^−16^
regulation of RNA metabolic process	90	2.80×10^−16^
skeletal system development	37	8.50×10^−16^
embryonic organ development	28	3.50×10^−15^
embryonic organ morphogenesis	25	7.70×10^−15^
neuron differentiation	39	4.90×10^−13^
anterior/posterior pattern formation	23	3.00×10^−12^

Top ten biological functions for brain hyper-methylated adult-only sites not overlapping bivalent genes		
GO Term (biological function)	# of genes	Benjamini *P* value
immune response	84	3.00×10^−02^
cell proliferation	59	2.00×10^−02^
negative regulation of cell proliferation	51	1.80×10^−02^
defense response	74	4.10×10^−02^
regulation of cell proliferation	89	5.10×10^−02^
intracellular signaling cascade	128	1.10×10^−01^
regulation of protein transport	21	1.30×10^−01^
response to drug	32	1.40×10^−01^
regulation of apoptosis	87	1.40×10^−01^
regulation of programmed cell death	87	1.80×10^−01^

## Discussion

Age-associated DNA hyper-methylated sites in blood have been shown to overlap bivalent chromatin domains [3], which involve genes with functions in development and cellular differentiation [10]. Here we have confirmed the occurrence of such an overlap in additional datasets from blood, and revealed that genomic regions associated with age-related changes in DNA methylation in the human brain are also enriched in bivalent domains, suggesting that this phenomenon is likely ubiquitous, occurring across different tissue types.

Regarding the relationship between brain age-associated DNA methylated sites and bivalent domains, we noted two primary trends: (1) that overlap of these sites was largely driven by DNA hyper-methylation, as hyper-methylated sites were more enriched in bivalent domains than hypo-methylated sites; and (2) that this enrichment was more pronounced for sites associated with DNA hyper-methylation that occurred in adulthood. Numata et al. [8] noted that DNA hyper-methylated sites represented a larger proportion of age-associated changes in adults compared to children, and discussed the fact that such trends may be consistent with the observation that stochastic changes in DNA methylation are known to increase with age [16]. Importantly, stochastic changes in DNA methylation have also been implicated in disease discordance between monozygotic twins [17], [18], including Alzheimer's disease [18].

But why DNA hyper-methylated sites show preferential overlap with bivalently marked genes, and whether these sites have roles in disease pathogenesis remains to be determined. Rakyan et al. [3] posited that increased DNA hyper-methylation at bivalent domains with age may be associated with a decrease in cellular and developmental pluripotency, in line with observations from *in vitro* studies using human cell lines [19]. Interestingly, with respect to the brain data analyzed here, we found that a subset of genes characterized by both age-associated DNA hyper-methylation and bivalent chromatin marks were enriched for the biological function “neuron differentiation”. Under a model such as that proposed by Rakyan et al. [3], and considering the two primary trends noted above, it could be speculated that the accumulation of methylation (typically associated with decreased gene expression) at bivalent loci in the aging brain could prevent neuronal cell regeneration. Thus, given that one of the primary pathologic features of age-related neurological disorders such as Alzheimer's disease and Parkinson's disease includes the loss of neuronal cell plasticity and cell death [20], [21], the relationship between the epigenetic alterations investigated in this study could have involvement in the onset or the progression of such disorders, and warrants further study.
